# Cigarette smoking is associated with plasma Aβ levels decreasing: a population based cross‐sectional study

**DOI:** 10.1002/alz70856_098178

**Published:** 2025-12-24

**Authors:** Chen Chen, Liangjun Dang, Ling Gao, Jie Liu, Shan Wei, Suhang Shang, Jin Wang, Jingyi Wang, Fan Gao, Qiumin Qu, Yongning Deng

**Affiliations:** ^1^ The First Affiliated Hospital of Xi'an Jiaotong University, Xi'an, Shaanxi, China; ^2^ The First Affiliated Hospital of Xi’an Jiaotong University, Xi'an, Shaanxi, China; ^3^ The First Affiliated Hospital of Xi'an Jiaotong University, Xi’an, Shaanxi, China; ^4^ The First Affiliated Hospital of Xi'an Jiaotong University, Xi’an, China; ^5^ Huxian Hospital of Traditional Chinese Medicine, Xi'an, Shaanxi, China

## Abstract

**Background:**

Growing evidence suggested cigarette smoking is a potential risk for Alzheimer's disease (AD), however, the underlying mechanism had not been clarified. As amyloid‐β (Aβ) deposition in the brain is the main pathophysiology of AD and plasma Aβ is closely related to Aβ accumulation in the brain, in the present study we investigated the relationships between smoking and plasma Aβ levels in a community population.

**Method:**

The population was selected using clustering sampling method and people who lived in the Qubao village of Xi’an aged 40 years or older were all enrolled. A face‐to‐face survey was used to assess the general information and smoking situation from October 8, 2014, to March 30, 2015. ELISA was used to measure the plasma levels of Aβ_1‐40_ and Aβ_1-42。_The relationship between smoking and plasma levels of Aβ was analyzed by multiple linear regression.

**Result:**

A total of 1464 subjects were enrolled in this study, and there were 414 smokers (28.3%). The plasma levels of Aβ_1‐40_, Aβ_1‐42_ and Aβ_1‐40_/Aβ_1‐42_ ratio had no significant differences between smoking group and non‐smokers group (*p* > 0.05). However, plasma Aβ_1‐40_ level was lower in the short‐term smoking group than that in the long‐team smoking group (49.92±10.29 pg/ml vs. 52.83±9.34 pg/ml, *p* <0.05). In the multiple linear regression analysis, plasma Aβ_1‐40_ level related with short‐term smoking negatively (*r* = ‐3.49, *p* <0.05), but Aβ_1‐42_ did not (*p* > 0.05). In the age‐stratified analysis, in the ≤65 years group, short‐term smoking group had lower plasma Aβ_1‐40_ (r=‐3.959, *p* < 0.05) and Aβ_1‐40_/Aβ_1‐42_ ratio (r=‐0.153, *p* < 0.05), while in the >65 years group, Aβ_1‐42_ was lower in the long‐term smoking group than that in the non‐smoking group (r=‐3.856, *p* < 0.05).

**Conclusion:**

Smoking was associated with decrease of plasma Aβ level, but the effects of smoking on plasma Aβ levels were affected by the smoking duration and age.

